# Therapy and Management of *Pneumocystis jirovecii* Infection

**DOI:** 10.3390/jof4040127

**Published:** 2018-11-22

**Authors:** P. Lewis White, Jessica S. Price, Matthijs Backx

**Affiliations:** Public Health Wales Microbiology Cardiff, UHW, Heath Park, Cardiff CF14 4XW, UK; jessica.price2@wales.nhs.uk (J.S.P.); Matthijs.backx2@wales.nhs.uk (M.B.)

**Keywords:** *Pneumocystis* pneumonia, PcP therapy, PcP diagnosis

## Abstract

The rates of *Pneumocystis* pneumonia (PcP) are increasing in the HIV-negative susceptible population. Guidance for the prophylaxis and treatment of PcP in HIV, haematology, and solid-organ transplant (SOT) recipients is available, although for many other populations (e.g., auto-immune disorders) there remains an urgent need for recommendations. The main drug for both prophylaxis and treatment of PcP is trimethoprim/sulfamethoxazole, but resistance to this therapy is emerging, placing further emphasis on the need to make a mycological diagnosis using molecular based methods. Outbreaks in SOT recipients, particularly renal transplants, are increasingly described, and likely caused by human-to-human spread, highlighting the need for efficient infection control policies and sensitive diagnostic assays. Widespread prophylaxis is the best measure to gain control of outbreak situations. This review will summarize diagnostic options, cover prophylactic and therapeutic management in the main at risk populations, while also covering aspects of managing resistant disease, outbreak situations, and paediatric PcP.

## 1. Introduction

The incidence of *Pneumocystis* pneumonia (PcP) is rising as a result of an increase in the susceptible patient population. This has occurred outside of the usual HIV-positive cohort, with data from recent studies showing that PcP is now more prevalent in the HIV-negative at-risk population [1,2,3,4,5,6]. Susceptible non-HIV infected patients include those with solid tumours, recipients of solid organ transplant (SOT), particularly renal transplants, and patients suffering from haematological malignancy, especially those receiving a haematopoietic stem cell transplant or those with a lymphoproliferative condition [6]. Although cases in renal transplant recipients exceed those in other SOT recipients, rates of infection are greatest in heart, lung, and combined heart-lung recipients [7]. With more patients receiving immune-modularity therapies (e.g., high dose corticosteroids or anti-TNF therapy) for autoimmune and inflammatory conditions and improvements in the recognition of primary immune deficiencies, the at-risk population continues to expand, with PcP being diagnosed in patients considered, typically, at lower risk (Table 1) [8,9].

The historical difficulties in diagnosing PcP has resulted in many cases being treated on clinical suspicion alone. In a recent 10-year audit of PcP in the UK, reported deaths due to PcP regularly exceeded the number of laboratory confirmed cases. With the advent of modern diagnostic techniques (Real-time PCR and (1-3)-β-d-Glucan (BDG)) this discrepancy should be reconciled to some degree [1]. PcP in HIV-negative patients often presents non-specifically and follows a more fulminant course. Since timely treatment improves prognosis, clinicians should commence antimicrobials based on clinical suspicion whilst awaiting the results of mycological investigations. PcP is associated with the significant morbidity and mortality, but remains relatively uncommon across most at-risk groups (<5%) [3,4,5]. In HIV-positive patients PcP usually presents as a sub-acute progressive deterioration over a period of weeks, whereas in HIV-negative patients an acute presentation over a few days is more typical [7]. PcP severity can be stratified into mild, moderate or severe disease, depending on presenting symptoms, oxygen saturation, and chest radiology (Table 2) [19]. Extra-pulmonary disease is unusual. The classification of disease severity is applicable regardless of the patient’s predisposing underlying condition [19,20]. Symptoms are generally non-specific, typically pneumonia, other symptoms, include fever, non-productive cough, worsening chest pain, shortness of breath (especially on exertion). Low arterial-oxygen tension may lead to respiratory failure, requiring mechanical ventilation and vasopressor, which is a poor prognostic feature.

Risk factors associated with PcP are well described (e.g., CD4 count <200/µL, graft versus host disease, corticosteroids), but are continually amended to reflect advances in medical therapies [7,21,22]. A meta-analysis of risk-factors associated with increased mortality from PcP include age, sex, delay in diagnosis, respiratory failure, solid tumours, high lactate dehydrogenase, low serum albumin, and bacterial, *Aspergillus* or Cytomegalovirus co-infection [23]. A range of co-infections associated with PcP is shown in Table 3.

A study evaluating PcP in Spanish HIV-positive patients confirmed the reduction in the incidence of PcP, but also showed a reduction in the rates of mortality [34]. It identified associated colder climatic conditions and higher levels of air pollutants as risk factors for PcP and mortality. However, evidence linking environmental conditions with PcP is conflicting [35,36]. Various prognostic markers have been determined in both HIV-positive and HIV-negative cohorts [37,38].

Using the various guidelines this review will summarize diagnostic options, cover prophylactic and therapeutic management in the main at risk populations, while also covering some specific, recent considerations for the management of PcP [39,40,41,42,43,44].

## 2. Diagnosis

**Figure 1:**
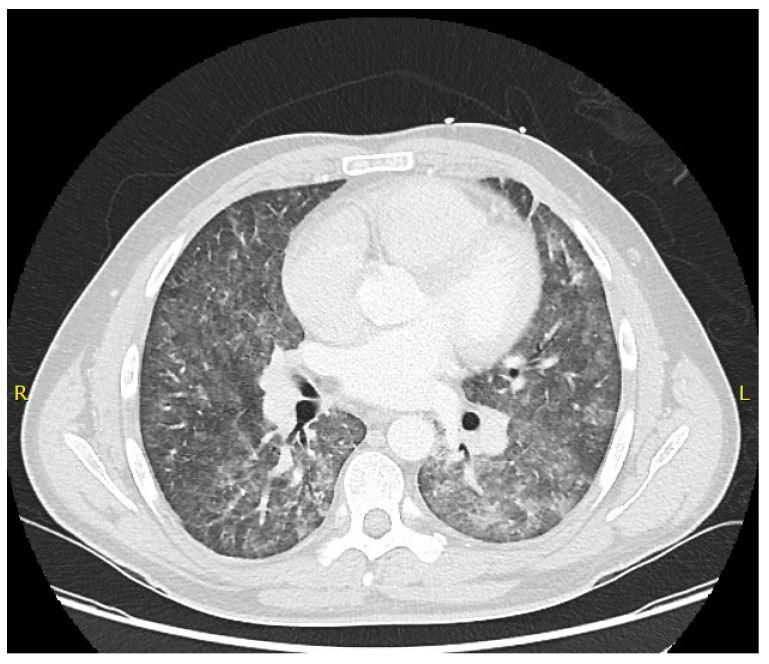
A computerized tomography (CT)-Thorax image showing centrilobular ground glass opacification in an HIV-infected patient with *Pneumocystis* pneumonia.

Computerized tomography (CT) is a sensitive modality for the diagnosis of PcP and should be considered in the early stages of disease when chest X-rays may be normal [45]. Using CT, early PcP can present with diffuse ground glass shadowing (GGO) (Figure 1), which progresses to GGO and patchy consolidation, with predominant consolidations in the latter stages of disease [45]. Other findings can occur, including nodules, pneumothoraces and rarely cavities [21]. The aforementioned radiological findings are not specific to PcP and are therefore not pathognomonic. A normal CT may be useful for excluding PcP [46].

Non-mycological laboratory investigations may prove beneficial. Elevated lactate dehydrogenase (LDH > 500 mg/dL) is associated with lung damage, and may be a useful adjunct test in the HIV-positive patient, where high concentrations may indicate severe disease [47]. If levels are normal it can be used to exclude PcP, but limited specificity requires any elevated level to be confirmed with a mycological assay [48]. The use of LDH in the HIV-negative population appears limited, with poor sensitivity and specificity. Procalcitonin levels are raised during pneumonia and levels associated with PcP are comparable to other pathogens capable of causing atypical pneumonia (e.g., *Mycoplasma* sp.) However, levels associated with PcP are significantly lower than those encountered with bacterial pneumonia and tuberculosis [49]. C-reactive protein may be normal in PcP and can therefore not be used to rule out infection [47]. The development of highly sensitive, non-culture based tests have enabled laboratories to diagnose PcP more readily. Although axenic culture of *Pneumocystis* has proven possible, its applicability to a routine diagnostic laboratory is minimal [50]. Microscopy is useful for confirming a diagnosis, and immuno-fluorescent (IF)-microscopy using fluorescently labelled monoclonal antibodies targeting both ascus and trophic forms is recommended [41]. IF-Microscopy performance is more accurate when testing deeper respiratory samples (e.g., bronchoalveolar lavage (BAL) fluid) as compared to superficial samples such as sputum [51].

PcP PCR provides enhanced sensitivity over conventional methods, with meta-analyses demonstrating a sensitivity of ≥97% and negative predictive value (NPV) ≥ 99% [52,53,54]. While there are concerns over the detection of *Pneumocystsis* colonization, the positive predictive values are good (given the low prevalence of the disease) and confirmed by the positive likelihood ratios (≥10). A negative PcP PCR on BAL fluid can be used to exclude disease. Positivity when testing upper airway samples was once thought to represent detection of colonization; it likely reflects a significant burden lower in the respiratory tract. There is no requirement for nested-PCR assays and conventional PCR should be replaced with real-time assays that can be used to quantify burden to determine clinical significance and differentiate colonization from infection. The significance of lower levels of PcP, as detected by PCR, need to be interpreted in conjunction with the clinical context in which the sample was obtained and the quality of the sample on which the assay was performed. The availability of commercial real-time assays will help address the current lack of assay standardization [55].

The detection of (1-3)-β-d-Glucan (BDG) in serum/plasma allows PcP to be confidently excluded if negative, with meta-analyses demonstrating a sensitivity and NPV of ≥ 90% and ≥97%, respectively [56,57,58]. BDG is not specific to PcP and can positive as a result of other fungal pathogens to which these patients may be susceptible. Although BDG concentrations are typically high in patients with PcP (> 500 pg/mL), low positive concentrations may occur [59]. BDG has been shown to differentiate PcP from *Pneumocystis* colonization, with BDG levels associated with latter generally below <90 pg/mL, while with disease levels were >100 pg/mL [59]. Although BDG testing of BAL fluid has been performed, it adds little to the testing of serum, with reduced specificity as a result of the presence of respiratory tract commensal organisms, such as *Candida* species or non-*Pneumocystis* colonizers [60,61]. Positive BDG concentrations have been documented in haematology patients and those undergoing haemodialysis in the absence of fungal disease, and needs to be taken in to consideration when evaluating test results in these cohorts [62].

Combination testing (IF-Microscopy with PcP PCR and BDG) has been proposed for the optimal diagnosis of PcP in the haematology setting. It is reasonable for this strategy to be applied to other cohorts, particularly HIV-positive patients, where the fungal burdens are often greater, simplifying detection [41]. The high sensitivity of BAL fluid PcP PCR and serum/plasma BDG may obviate the need for IF-microscopy, particularly if the PCR test is quantifiable. However, given the current lack of standardization of both BAL fluid sampling and PCR amplification, IF-microscopy does enhance specificity when positive. Sensitive molecular and biomarker assays are detecting patients with minimal symptoms or who are asymptomatic. Clinically, where a symptomatic patient is BDG and PcP PCR positive but microscopy negative, it is likely that the patient has PcP. IF-Microscopy remains the gold standard for the diagnosis of PcP, but guidelines recognize the utility of both PCR on BAL fluid and BDG on serum, where negativity can be used to exclude infection [39,41,42,44]. This will likely change once PCR is standardized and PCR cycle threshold values (Cq) are correlated to a burden equating to positivity by IF-Microscopy [41].

## 3. Primary Prophylaxis

Prophylaxis of at risk patients is considered a mainstay for the prevention of PcP. However, with a rapidly-growing and diverse at risk population certain patient populations may not receive the necessary prophylaxis, due to the absence of guidelines and current limitations on the knowledge of risk. This includes patients receiving disease modifying drugs and aggressive chemotherapeutic regimens for an array of inflammatory and malignant diseases; patients receiving TNF blockade (infliximab, adalimumab, and etanercept), CD-52 antibodies (alemtuzamab), calcineurin inhibitors (cyclosporin and tacrolimus), B-cell blockade (Rituximab) and selective T cell blockade therapies, in addition anti-purine drugs, bendamustine, nucleoside analogues, and high-dose steroids for prolonged periods [63,64,65,66].

Systematic review and meta-analysis has shown significant benefit in preventing PcP and reducing PcP related mortality, although individual trials primarily focusing on haematology and solid organ transplant patients and tended to be uncontrolled with limited numbers, impacting quality [66]. A variety of different prophylactic regimens have been used (Table 4) and the optimum regimen in many patient groups has not been determined. In general, trimethoprim/sulfamethoxazole (TMP/SMX) remains the drug of choice for prophylaxis, with second line choices considered to provide inferior protection, albeit with potentially fewer side-effects. An overview of prophylactic choices and administration is shown in Table 4.

### 3.1. HIV-Positive Population

In HIV-positive patients, PcP prophylaxis is recommended when CD4 counts are less than 200 cells/mm^3^ or if previously diagnosed with oropharyngeal candidiasis [39]. Prophylaxis should also be considered in patients previously diagnosed with an AIDS defining illness or if the CD4 cell percentage is less than 14%. If it is not possible to perform regular CD4 counts, then prophylaxis should be initiated when counts are between 200–250 cells/ mm^3^. The primary prophylactic agent is one single-strength TMP/SMX (80 mg TMP/400 mg SMX) daily or one double strength tablet (160 mg TMP/800 mg SMX)/daily [39]. Alternative prophylaxis includes one double strength TMP/SMX tablet three times per week, dapsone alone or in combination with pyrimethamine and leucovorin, pentamidine aerosols or atovaquone (Table 4).

It is recommended that prophylaxis in adults and adolescents be continued until CD4 T-cell counts are sustained at > 200 cells/mm^3^ for more than 3 months, but CD4+ cell percentage of ≥14%, and a sustained undetectable HIV plasma RNA levels can also be considered [39]. The European Collaboration of Observational HIV Epidemiological Research in Europe (COHERE) study provided evidence that it may be safe to cease prophylaxis in HIV-positive patients with a suppressed viral load and a CD4 count between 100-200 cells/mm^3^, reducing pill burden, drug toxicity and interactions, inconvenience and cost, and the potential development of bacterial resistance. [67] Prophylaxis should be recommenced if the CD4 count decreases to < 200 cells/mm^3^ [39]. If PcP was diagnosed when the patient’s CD4 count was >200 cells/mm^3^ life-time prophylaxis could be considered [39].

Prophylaxis for PCP during pregnancy is the same as for other adults and adolescents, with the caveat that prophylaxis might be withheld during the first trimester due to theoretical concerns regarding teratogenicity associated with drug exposures [39]. Due to the absence of systemic absorption and limited risk to the embryo, aerosolized pentamidine may be considered.

### 3.2. Solid Organ Transplant Recipients

In SOT recipients PcP prophylaxis is recommended for patients where the local incidence of disease is ≥ 3% [44,68]. The risk is greatest during the first six months’ post-transplant, where rates of 5–15% have been documented [7]. The duration for prophylaxis ranges from 3 months to > 1 year, but is generally 6–12 months. However, prolonged risk periods are likely in patients suffering from graft rejection, CMV infection (both chronic or recurrent), prolonged neutropenia, autoimmune disease or those treated with prolonged corticosteroid therapy (> 20 mg/day > 2 weeks) [22,44]. A minimum of six weeks’ prophylaxis has been recommended during and post therapy for renal graft rejection [69]. Lifelong prophylaxis may be considered in lung, heart, and small bowel transplant recipients, or those with a prior diagnosis of PcP or chronic CMV. Lifelong prophylaxis may also be considered in renal transplant recipients due to the frequent occurrence of PcP outbreaks in this setting [70]. While short-term prophylaxis is satisfactory for controlling an individual outbreak, the contagious nature of PcP and prolonged risk period of these patients may warrant the additional prophylaxis to prevent further outbreaks. The exact dose of TMP/SMX used for life-long prophylaxis is unclear as hyperkalaemia, a result of graft dysfunction, may necessitate dose adjustment [70]. Attempts to stratify the need for additional PcP prophylaxis in SOT patients have been made [7,71]. A recent study evaluated risk factors 6 months post renal transplantation and multivariate analysis showed that induction therapy mediated by anti-thymocyte globulin, steroid therapy, high doses of calcineurin inhibitors (tacrolimus ≥ 0.5 mg/kg/day and cyclosporine ≥ 2.1 mg/kg/day) and CMV were associated with late PcP [71].

As with PcP in HIV-positive individuals the primary prophylactic choice is oral or intravenous TMP/SMX, one single-strength (80 mg TMP/400 mg SMX)/day or double strength tablet (160 mg TMP/800 mg SMX), although prophylaxis can be administered daily or three times per week [44]. The efficacy of the double strength appears similar whether daily or thrice weekly, but daily administration is required to protect against other post-transplant infections (e.g., Toxoplasmosis) [22,44]. A meta-analysis showed that the risk reduction using TMP/SXM prophylaxis in SOT (0.09, 95% CI: 0.02–0.48) was similar to that for the overall HIV-negative at risk population (0.15, 95% CI: 0.04–0.62) and adverse events were rare [72]. Oral dapsone (50–100 mg once a day or 100 mg weekly) may be used as a second-line prophylactic agent, although intolerance to TMP/SMX increases the likelihood that this will occur with dapsone [22,44]. Once weekly dapsone prophylaxis has been successfully used to prevent PcP in renal and liver transplant patients with contraindications or intolerance to TMP/SMX [73]. While there were no cases of PcP in the dapsone or TMP/SMX arms, there were more breakthrough (mainly bacterial) infections and more hospitalized patients when using dapsone prophylaxis [73]. Intolerance to dapsone can arise more regularly in SOT recipients [74]. Oral atovaquone has also been used as an alternative prophylaxis in SOT. While it as effective as dapsone in HIV-positive, there is less evidence in SOT patients and an optimal dose has not been determined, but is likely >1000 mg (likely 1500 mg) daily [22,44]. Mutations in *Pneumocystis* have been associated with failure of atovaquone prophylaxis [75,76]. In a recent study, a mutation in the mitochondrial Cytochrome *b* gene possibly altered the atovaquone binding pocket, leading to prophylaxis failure and a PcP outbreak in a cardiac transplant unit. [77] Inhaled (nebulized) pentamidine (300 mg every 3–4 weeks) is considered a third-line agent, requiring experienced staff to administer the drug and has been associated with higher rates of break-through infections and disseminated disease [22,44]. While there is anecdotal evidence of combination approaches and other routes of administration the evidence limited.

### 3.3. Haematology Patients

Comprehensive recommendations for PcP prophylaxis for patients with haematological malignancies and those undergoing SCT have been made by the European Conference of Infections in Leukaemia (ECIL) [43]. PcP prophylaxis should be commenced in acute lymphoblastic leukaemia patients, allogeneic stem cell transplant recipients, patients being treated with alemtuzumab, fludarabine/cyclophosphamide/rituximab combinations and patients on prolonged (≥20 mg/day >4 weeks) corticosteroids treatment. Additional indications include lymphoma patients receiving R-CHOP14 or escalated BEACOPP, patients receiving alternative lymphocyte depleting compounds (e.g., nucleoside analogues), or radiotherapy for brain tumours/metastasis in conjunction with steroid therapy [43]. Evidence is lacking for patients diagnosed with acute myeloblastic leukaemia, cerebral lymphoma, certain lymphoproliferative disorders, autoimmune cytopenia and haemophagocytic syndromes. The introduction of immune-modulators (anti-TNF-α) and delayed T-cell recovery post transplantation (e.g., cord blood transplants/haplo-identical transplants) will likely increase the haematology population at risk of PcP [43].

The recommended primary prophylaxis is TMP/SMX one single-strength tablet (80 mg TMP/400 mg SMX)/day or one double strength tablet (160 mg TMP/800 mg SMX)/day or thrice weekly, but this recommendation has not been fully validated in a randomized control trial. [43] Secondary prophylaxis includes dapsone, pentamidine aerosols, or atovaquone (Table 4). Again, no randomized control trials have been performed to compare performance or determine optimal dosage. In a large retrospective cohort study of primary prophylaxis in stem cell transplant recipients, aerosolized pentamidine had the highest probability of PcP but lower probability of toxicity, although another study showed higher rates of toxicity [78]. While rates of breakthrough PcP in stem cell transplant recipients receiving dapsone have been shown to be higher than those receiving TMP/SMX, rates are lowered if dapsone is administered daily compared to thrice weekly, although rates remain greater than when using TMP/SMX [79]. A randomized control trial of atovaquone versus TMP/SMX as primary prophylaxis in autologous stem cell transplant recipients resulted in no cases of PcP in either arm, but more intolerance to TMP/SMX [80]. The use of these second-line agents removes any protection from toxomplasmosis and bacterial infections, and may require specialized equipment and training (pentamidine), additional drug costs (atovaquone), and may result in other toxicities [43].

There is no definitive evidence on when to commence, or the duration of PcP prophylaxis in the haematological population. Initiation of prophylaxis during the pre-engraftment period in HSCT recipients (with the possible exception of the conditioning period) is not recommended, due to the potential for marrow toxicity when using TMP/SMX. Primary prophylaxis is recommended for the period of treatment-induced immunosuppression or until the CD4+ cell count increases to >200 cells/mm^3^.

In allogeneic stem-cell recipients, prophylaxis from engraftment until ≥6 months after transplant is recommended, but longer may be necessary in patients receiving continued immunosuppressive drugs and/or in those that have chronic graft versus host disease [43]. In patients receiving alemtuzumab or fludarabine/cyclophosphamide/rituximab combinations prophylaxis is recommended for at least six months’ post completion of treatment. Unlike in patients living with HIV, there is no robust evidence supporting stopping prophylaxis when the CD4+ cell count >200 cells/mm^3^. This is likely due to the presence of additional risk factors, such as the use of steroids to manage GVHD [43].

### 3.4. Other Populations

There is a need for guidance regarding PcP prophylaxis for several cohorts outside the aforementioned groups. In particular, patients with rheumatological and other autoimmune diseases are receiving more intense therapy for management of their underlying conditions, resulting in impairment of cell-mediated immunity that increases risk of PcP, but knowledge on individual risk factors and predictors of PcP is sparse. A recent attempt to evaluate the incidence and risk factors for the development of PcP in patients diagnosed with connective tissue disorders confirmed the limited availability of data and subsequent exclusion of this cohort from systematic reviews [81]. Currently, evidence based recommendations specific to rheumatology cannot be provided, but prophylaxis should be considered in patients receiving intense immunosuppressive therapy, particularly in individuals with lymphopenia and a low CD4 count. Similar prophylaxis should be used in patients with ANCA-positive vasculitis (granulomatosis with polyangiitis) undergoing intense induction therapy [81]. Risk factors for PcP in rheumatology include older age (>65 years old), coexisting pulmonary disease and the use of glucocorticoids [82]. When initiating prophylaxis in patients with two or three risk factors for PcP the incidence of PcP was reduced to 0.00 from 0.93 per 100 person-years prior to implementation, with no severe adverse events associated with TMP/SMX treatment. [82] In a second large study investigating the efficacy and safety of TMP/SMX as primary prophylaxis for PcP in rheumatology patients receiving high-dose steroids, the incidence of PcP and related mortality after 1 year was reduced using prophylaxis. Prophylaxis was well tolerated with the number needed to treat to prevent one case of PcP (52, 95% CI:33–124) lower than the number needed to harm through serious adverse event (131, 95% CI:55–∞) [65]. Although CD4 counts play a significant role in determining risk from PcP in other cohorts (HIV, Haematology, and SOT), its role in autoimmune and inflammatory disease is less well defined, and the median CD4 count in PCP patients with this syndrome was 302/mm^3^ compared to 19/mm^3^ in HIV-positive patients [83].

An evaluation of PcP prophylaxis in patients treated with systemic corticosteroids, or other immunosuppressive agents, for immune-mediated dermatologic conditions indicated there was no support for routine administration of universal PcP prophylaxis in patients taking immunosuppressive medications, with each case requiring individual consideration [84]. There have also been calls for guidance on PcP prophylaxis in patients suffering from endocrine conditions, such as Cushing’s syndrome and patients being treated for inflammatory bowel disease [17,85].

## 4. Therapy

Trimethoprim, sulfa drugs, and pentamidine form the main stays of treatment. Irrespective of underlying condition, the first-line therapy for PcP is TMP/SMX. Alternative regimens may depend on the severity of disease and the underlying condition, with these factors also determining whether corticosteroids are used. Despite *Pneumocystis jirovecii* being classified in the fungal kingdom on the basis of DNA sequence similarity and cell wall composition, it does not contain ergosterol in the cell wall. Consequently, polyenes (Amphotericin B/liposomal Amphotericin) or azoles (fluconazole, itraconazole, voriconazole, posaconazole, and isavuconazole) antifungals cannot been used for prophylaxis or treatment. The different morphological forms also show varying susceptibility to echinocandins (e.g., caspofungin) with in vitro inhibition of the cyst but not trophic forms [86]. An overview of treatment choices and administration is shown in Table 4.

### 4.1. HIV-Positive Population

The recommended treatment for PcP in HIV is intravenous TMP/SMX with dose adjustment required in renal dysfunction [39]. For mild-moderate disease oral administration may be considered. Corticosteroids are of proven benefit in HIV-infected individuals with severe disease. A meta-analysis on the use of steroids for PcP in HIV-positive patients reported that their use was associated with reduced mortality, particularly in the early phase of disease [87]. In moderate to severe disease corticosteroids should be initiated within 72 h.

In mild/moderate disease alternative second-line therapies include dapsone/trimethoprim, primaquine (oral only) plus clindamycin (IV) and atavquone suspension, although the latter is less effective than TMP/SMX, and dapsone/trimethoprim has better efficacy while limiting side-effects [39]. Second-line therapy for moderate/severe disease includes intravenous pentamidine or clindamycin/primaquine, the latter being better tolerated although less efficacious. Aerosolized pentamidine is not recommended for treatment [39].

Treatment duration is three weeks, but longer may be necessary, dependent on disease severity, drug choice, immunodeficiency, previous infection and early initiation of therapy. Mechanical ventilation and ICU care can be used, dependent on functional status [39]. Improvement should be expected within 4–8 days, but without steroids it is not unusual to see a deterioration in the first 3–5 days, likely associated with an inflammatory response. Investigations (BAL fluid) for concomitant respiratory infections (See Table 4) should be performed if there is no clinical improvement or worsening symptoms. Changing to parenteral pentamidine or clindamycin/primaquine is recommended if there is TMP/SMX toxicity or treatment failure [39].

The most effective way of preventing PcP in people living with HIV is by immune-reconstitution, through the administration of effective anti-retroviral therapy. Additive or synergistic toxicities associated with anti-PcP and antiretroviral therapies, may lead to a delay in the initiation of antiretroviral therapy until after initiating anti-PCP therapy or, in some cases, until after the completion of anti-PcP therapy. However, a large multi-centre trial of opportunistic infections in 282 newly diagnosed HIV patients compared immediate anti-retroviral therapy with therapy delayed until completion of treatment for the opportunistic infection, supported the initiation of anti-retroviral therapy alongside PcP treatment [88]. Immediate initiation of both therapies was not associated with increase in side-effects or a reduction in the efficacy of anti-retroviral therapy but was associated with a reduction in AIDS progression and mortality rates. The risk of immune reconstitution inflammatory syndrome (IRIS) in PcP/HIV-positive patients who are treated for both infections has been deemed low [47]. In the previous multicenter trial, the rates of IRIS were 5.7% and 8.5%, for immediate treatment of opportunistic infection and HIV versus deferred antiretroviral therapy, respectively [88].

### 4.2. Solid Organ Transplant Recipients

Daily intravenous TMP/SMX (15–20 mg/kg TMP; 75–100 mg/kg SMX) is the most efficacious treatment for mild to severe PcP in SOT [22,44]. A recent study compared performance of low dose trimethoprim (<15 mg/kg/day) with the conventional dose (15–20 mg/kg/day) in combination with sulfamethoxazole for treatment of PcP in SOT recipients [89]. There was no difference in mortality rates between the groups (*p* = 0.76), but adverse effects were significantly reduced with the lower dose (42% compared to 17%; *p* = 0.02). As with HIV-positive patients, IV pentamidine is the preferred second-line agent, although for pancreas and islet cell transplants alternatives to pentamidine should be sought to avoid potential pancreatic dysfunction and islet cell necrosis [44]. Oral TMP/SMX has excellent bioavailability and may be considered in patients with mild-moderate disease [22]. It is recommended to reduce immunosuppression when managing PcP in kidney transplant recipients [69].

Duration of therapy is a minimum of 14 days, up to 21 days for severe disease [44]. While many other PcP therapy combinations have potential for use in SOT, data is limited and evidence extrapolated from studies in the HIV-positive population. Patients usually show clinical improvement within 4–8 days, although, as with HIV-positive, deterioration can occur during the first few days of therapy [22].

In SOT patients with moderate to severe disease, twice-daily 40–60 mg prednisone for 5–7 days within 72 h of diagnosis has been recommended, with a gradual reduction in dose over a further 7–14 days in order to prevent immune reconstitution pneumonitis [44]. One study associated prolonged survival with the administration of steroids, but generally there is little evidence to support their use in SOT and the optimal dose is not known [22,90]. Retrospective small studies of heterogeneous populations provide conflicting findings, even associated with an increased mortality rate and a meta-analysis and systematic review did not support an association with steroid use and improved survival in SOT [91,92]. A retrospective cohort study of 323 HIV-negative patients showed the use of early steroids did not alter mortality, length of stay, ICU admission of the requirement for mechanical ventilation, but was associated with less improvement in the respiratory component of the sequential organ failure assessment score [93]. Given that corticosteroid use may be a risk factor for the SOT patient developing PcP, the use of such therapy as a treatment appears rather conflicting.

### 4.3. Haematology Patients

Recommendations for the treatment of PcP in haematology patients have been provided by ECIL and may be generally applicable to all on HIV-negative patients with PcP [20].

Again, IV TMP/SMX (15–20 mg/kg TMP; 75–100 mg/kg SMX) is the treatment of choice. Initiation should be prompt as disease is regularly severe in presentation and delays increase the need for intensive care and mechanical ventilation, leading to higher mortality. Treatment is recommended in at-risk patients with clinical signs, including dyspnoea, cough, fever, hypoxaemia, and chest pain, in the presence of typical chest radiology (preferably Chest CT) and an elevated, yet unexplained, serum LDH concentration [20]. Pulmonary toxicity associated with therapies used to treat the underlying condition can present in manner that resembles PcP. Nevertheless, an attempt to achieve a diagnosis should be prompt, but should not delay initiation of treatment, particularly as bronchoscopy is often contraindicated in this patient population. This highlights the difficulty in managing these patients and the importance of diagnostic approaches testing non-invasive samples such BDG on serum or PCR on upper airway samples [41].

Duration of therapy is a minimum of 14 days, with three weeks usually required for severe or non-responsive cases. As with SOT recipients, haematology patients with PcP can be slow to respond and may deteriorate clinically in the first few days of treatment. Assessment of treatment failure cannot be made confidently during the first week of treatment. If there is no clinical improvement after eight days of therapy, treatment failure should be suspected and repeat investigations (e.g., Bronchoscopy and chest CT) should be performed to identify co-infections, which can occur in approximately one in five patients (Table 3) and PcP complications (e.g., pneumothorax) [91,94]. The use of serial BDG monitoring to determine clinical response is not recommended. Persistent PcP PCR positivity in repeat BAL fluid is not suggestive of treatment failure as this may indicate prolonged persistence of the organism in the respiratory tract [20,95].

Recommended second-line therapy includes IV pentamidine (4 mg/kg/day), oral atovaquone (750 mg/2–3 per day), or oral primaquine (30 mg/day) plus IV or oral clindamycin (600 mg/3 per day) [20]. The efficacy of second-line regimens is not well validated and should only be used in severe intolerance, or when primary treatment failure is documented in the absence of other causes (e.g., co-infection). Primaquine/clindamycin is the preferred second-line option [20].

Prompt intensive care support is recommended for haematology patients with PcP as there is a high incidence of acute respiratory failure and an associated increase in mortality rates with delays in transfer to critical care [20]. Non-invasive ventilation may be used as a primary strategy for hypoxic acute respiratory failure, but failure rates with PcP can be high [20,96]. Monitoring for non-invasive ventilation failure is essential, with poor tolerance, an unidentified cause of respiratory failure, clinical or respiratory deterioration, limited clinical and arterial blood gas improvement within six hours, persistent high respiratory rate (>30/min), prolonged (>3 days) dependency on non-invasive ventilation all indications for intubation and mechanical ventilation [20,97]. As with SOT recipients, adjunctive corticosteroids can be considered on a case to case basis, due to the heterogeneity and conflicting evidence of retrospective observational studies and absence of controlled trials in HIV-negative patients. Of 11 studies investigating corticosteroid use for PCP HIV-negative patients, including haematology, only four reported a reduced mortality rate when using corticosteroids, leading to the decision not to recommend the use of corticosteroids when treating PcP in haematology [20]. The fact that corticosteroids are widely used in the management of haematology malignancies and are in themselves a risk factor for PcP, complicates the administration of these drugs and highlights the need for controlled investigations.

## 5. Secondary Prophylaxis

HIV-positive patients diagnosed with PCP should be administered secondary prophylaxis with TMP/SMX until immune reconstitution is achieved [39]. In a randomized controlled trial in HIV-positive patients comparing the secondary prophylactic use of TMP/SMX versus inhaled pentamidine, the risk of PcP recurrence was 3.3 fold higher using pentamidine (*p* < 0.001) [98].

Second-line regimens include dapsone, dapsone combined with pyrimethamine, atovaquone, or aerosolized pentamidine [39]. All HIV-negative patients successfully treated for PcP should receive secondary prophylaxis following the same guidelines for primary prophylaxis. The duration of secondary prophylaxis in the HIV-negative has not been determined and should be judged on a case-by-case basis, following primary prophylaxis protocols [20].

## 6. Side-Effects and Interactions

Many of the drugs used to manage PcP are associated with adverse events with higher rates reported in haematology patients [43]. Adverse events with TMP/SMX are relatively frequent, and include rashes, fever, gastrointestinal upset, cytopenia, marrow suppression, electrolyte disorders (hyperkalemia), hepatoxicity, interstitial nephritis, aseptic meningitis, anaphylaxis, renal insufficiency and pancreatitis. These side effects occur more frequently when using therapeutic TMP/SMX doses and may require use of second-line agents [72]. Allergies to sulfa-based drugs are a contraindication to their use. TMP/SMX can alter levels of creatinine and cyclosporine. Although the use of secondary agents may be associated with less side-effects the rate of breakthrough cases using second-line prophylaxis can be higher than TMP/SMX [99]. When adverse events resolve, and in the absence of anaphylaxis, it may be possible to reintroduce TMP/SMX.

Dapsone can trigger methaemoglobinaemia and haemolytic anaemia in susceptible individuals and patients should be screened for glucose-6-phosphate dehydrogenase (G6PD) deficiency prior to use. Other serious side-effects include a potentially fatal idiosyncratic dapsone-hypersensitivity syndrome causing fever, skin rash, eosinophilia, and major organ dysfunction. Other side-effects include desquamation, neutropenia, anaemia, agranulocytosis, gastrointestinal upset, interstitial nephritis, and hepatitis [21]. Atovaquone is generally well tolerated and it is likely as efficacious as the other second line agents, although its use may be limited by higher drug costs. Common side-effects include rash and gastrointestinal issues. Absorption of atovaquone is improved by the presence of fatty foods in the gut, with diarrhea having a detrimental effect. To monitor the variable gastrointestinal absorption, it has been proposed that concentration in the blood should be assessed through therapeutic drug monitoring [100].

Inhaled pentamidine has the advantage that it is administered monthly, but requires a jet nebulizer and side-room facilities for effective and safe administration. Common side-effects associated with inhaled pentamidine include bronchospasm and cough. For both IV and oral administrations interaction with other nephrotoxic drugs (e.g., cyclosporine) has been noted, enhancing renal toxicity. Other side-effects include hypo/hyper glycaemia, insulin-dependent diabetes mellitus, pancreatitis, hepatitis, cardiac arrhythmia (Q-T prolongation), hypotension, bone-marrow suppression, and electrolyte disorders.

Clindamycin/primaquine have been associated with nausea and vomiting, *Clostridioides difficile* infection, neutropenia, and haemolysis in patients with glucose-6-phosphate dehydrogenase deficiency [20].

A range of drug-drug interactions has been reported, including other PcP medications and other antibiotic therapies (rifampicin, rifabutin, and macrolides). Of importance when treating PcP in HIV-positive patients is the reduction in atovaquone concentration associated with the anti-retroviral therapy efavirenz [101]. In a single centre open-label, parallel-sequence, pharmacokinetic study, approximately 50% of patients receiving efavirenz had lower atovaquone concentrations compared to those in patients not receiving antiretroviral therapy. Only 50% of patients obtained sufficient average treatment concentrations (>15 μg/mL), indicating that a dose of 750 mg BID may be insufficient for treating mild-moderate PcP. All co-medications should be scrutinized for interactions with PcP therapy [20].

## 7. Resistance

Resistant PcP disease has been documented for more than a decade but it is difficult to determine the scale of the issue due to the limitations of conventional microbiological approaches. Polymorphisms in the genes targeted by anti-PcP therapies are increasingly recognized as reason for resistant PcP (Table 5). Mutations in dihydrofolate recuctase (DHFR) and dihydropteroate synthase (DHPS) are commonly reported as inferring resistance to TMP/SMX [102]. The A144V mutation in cytochrome *b* has been associated with resistance to atovaquone, resulting in prophylaxis failure and a PcP outbreak in cardiac transplant recipients, but other cytochrome *b* mutations have been noted (Table 5) [77].

Identifying resistance may be complicated by the difficulty in culturing PcP which may preclude in vitro susceptibility testing. Equally, low fungal burdens can limit the efficiency of molecular techniques. Rates of mutation can vary and their association with treatment failure remains uncertain. In a German study, 63% and 75% of 128 PcP cases had the DHPS and DHFR genes sequenced, respectively [102]. There was no correlation between mutations in either gene and treatment failure. In a South American study, the rate of DHPS mutations in sulfa-prophylaxis-naïve adults was investigated, along with the effect of these mutations on patient management [106]. Mutations were detected in approximately 50% of patients, irrespective of underlying disease (HIV-positive and negative). The presence of mutations was associated with a longer requirement for mechanical ventilation, but was not associated with increased mortality. The high rates of mutation have not been replicated in other Latin American countries [106]. Inter-human transmission may be predictor for rates of DHPS mutations in PcP sulfa-naïve patients [106,109,110]. In a study across two European centres, rates of DHPS mutations were significantly higher in the French centre, possibly as a result of more stringent isolation of cases and more aggressive prophylaxis in Switzerland [109].

Molecular investigations to identify polymorphisms include sequencing and real-time PCR assays [102,111]. The development of a commercial real-time PCR for both the detection of organism and dihydropteroate synthase (DHPS) point mutations helps provide methodological standardisation, widespread access to such tests and rapid results [111]. While, the sensitivity (70%) and specificity (82%) of the PneumoGenius^®^ assay were less than would be expected from a PCR assay, the performance may have been influenced by disease classification that lacked mycological evidence and was based on risk factors, clinical presentation and response to therapy. The assay determined that 4.5% of samples contained a DHPS mutation likely inferring sulfa-resistance [111].

While, the presence of mutations is clinically concerning, there is no guidance on whether to change therapy when present and this decision should be made on an individual case basis. The presence of a mutation in a case failing to respond, or who is deteriorating, certainly supports the need to change therapy.

## 8. Outbreaks

PcP outbreaks are well documented, particularly in the SOT setting, especially in renal transplant recipients. Transmission is likely through person-to-person spread via airborne dispersal from PcP cases, prodromal patients, or asymptomatic carriers (including other at-risk patients, but possibly health-care workers) [112,113]. This transmission need not be direct, with infected hosts contaminating both proximal (within 1 m) and distal (8 m) environments around the patient [114]. Outbreaks caused by a single predominant strain indicate recent inter-human transmission or a common environmental source, rather than reactivation of historical infection [115]. Patients with heavy fungal burdens in BAL fluid (e.g., early Cq value by real-time PCR) could be “hyper-spreaders” and considered a greater infection control risk [114].

A meta-analysis and systematic review evaluated 30 PcP outbreaks that were globally distributed, but mainly limited to the adult population. [112] The largest outbreak involved 97 SOT cases and was spread across 23 centres making identifying a single source difficult [116]. Local transmission events were associated with asymptomatic carriers of *Pneumocystis* sharing clinical waiting rooms, including outpatient areas. Inter-hospital spread occurred due to the transfer of PcP infected patients. Again the de novo development of infection through patient-to-patient transmission, as opposed to reactivation of latent disease, was supported by phylogenetic analysis [112]. Air sampling of rooms occupied by patients with PcP have been shown to contain *Pneumocystis*, supporting infection control measures such as isolation of infected patients and the use of facemasks capable of filtering out microbes [117,118]. Formal recommendations for the management of PcP outbreaks are not available due to limited data, but recommendations for the control of other respiratory pathogens (e.g., Respiratory viruses) may be prove useful in the interim. The confounding factor in most outbreaks is the use of suboptimal, or the absence of PcP prophylaxis [112]. Effective control of outbreaks is gained through the administration of prophylaxis to prevent further cases [20]. Patients diagnosed with PcP should not share facilities with other immunocompromised patients [39]. Screening by molecular tests to identify asymptomatic carriers attending rheumatoid arthritis clinics has been performed to prevent inadvertent introduction and exposure, and could be applied to enhance infection control in other clinical scenarios [119]. The meta-analysis of Yiannakis and Boswell provides excellent guidance on outbreak identification, environmental monitoring, and infection control measures for managing known cases and out-patient environments, and procedures for managing contacts [112].

Although environmental conditions have been associated with increased rates of PcP, data is conflicting. Environmental sampling during outbreak investigation has proven limited, with only one study finding the same strain of *P. Jirovecii* in the clinical environment and the outbreak, albeit this was post occupation with an outbreak case [120]. The evidence for the environment being the source of PcP outbreaks is limited and much less convincing than that supporting human-to-human transmission [112]. Genotyping is limited by the inability to culture the organism, and it is difficult to successfully apply molecular techniques to low burdens that may be evident in HIV-negative PcP disease. While most outbreaks that utilize genotyping confirm a predominant strain and likely spread between individuals, it is not always the case. More than 60 genotypes of *P. Jirovecii* have been identified and it was initially proposed that 30% of PcP cases were infected with multiple types, hampering the ability to investigate transmission and clusters [121,122]. More recent research utilizing next generation sequencing deemed PcP was caused by a mixture of different genotypes and not a single strain, supporting this theory [123]. Conversely, geographically linked renal cohorts have been shown to be infected by a dominant strain [124].

## 9. Paediatrics

It is well known that children are exposed to *Pneumocystis* at an early age, yet in the immuno-competent host infection is generally asymptomatic, at worst presenting as a mild respiratory illness. PcP is a common AIDS-defining illness in paediatrics, with the highest incidence in the first year of life. There has been a reduction in rates during the anti-retroviral era, complemented with interventions to prevent mother-to-child HIV transmission and PcP prophylaxis [42]. Transmission is usually human-to-human, through inhalation of air contaminated with by PcP case, but intrauterine cases have been reported [125]. CD4 count and CD4 percentage remain the most significant risk factors. Sign and symptoms vary in both presence and severity, but typically include fever, tachypenia, dyspnea, hypoxia, and cough, while extra-pulmonary disease is rare [42]. Diagnosis is similar to that in adults and can be made on clinical grounds (CD4 <200 cells/mm^3^, low arterial O_2_, and raised LDH) combined with radiology (e.g., ground glass opacification), but definitive diagnosis requires the demonstration of the organisms in respiratory tissue or fluid, preferably using monoclonal IF antibody staining. PCR testing provides enhanced sensitivity, potentially at the expense of specificity. Diagnosis can be complicated by the difficulty in attaining deep respiratory samples in paediatrics, but BAL fluid remains the preferred specimen. Trans-bronchial biopsy has been recommended, if BAL fluid is inconclusive in cases with a typical clinical presentation, but this procedure has not been widely implemented due limited numbers of trained physicians [42].

As with PcP in the adult population TMP/SMX is the recommended choice for both prophylaxis and treatment. Prophylaxis is recommended for HIV-positive infants aged ≥ 6 years with CD4 < 200 cells/mm^3^ or CD4 < 15%, infants aged 1–5 years with CD4 counts <500 cells/mm^3^ or CD4 < 15% and all infants until 1 year of age irrespective of CD4 risk [42]. Infants born to HIV-positive mothers should receive prophylaxis from 4–6 weeks until HIV-infection can be excluded, at which point prophylaxis is not required. Trimethoprim (150 mg/m^2^ body surface area/day)/sulfamethoxazole (750 mg/m^2^ body surface area/day) prophylaxis can be administered orally, twice daily, or three times a week, with dose adjustment required for renal impairment, and total daily dose should not exceed trimethoprim (320 mg)/sulfamethoxazole (1600 mg) [42].

Oral atovaquone (dose dependent on age), oral dapsone (2 mg/kg/day or 4 mg/kg/week) and aerosolized pentamidine (300 mg/month) are alternative choices. IV pentamidine is not recommended. [42] Prophylaxis should continue in HIV-positive newly born infants for the first year of life, and if required until CD4 recovery. In older children prophylaxis duration is governed by recovery of CD4 count or percentage, usually requiring six months of anti-retroviral therapy initiation, with regular monitoring of CD4 levels to identify new period of risk.

For treatment of infants >2 months old, the dose is IV trimethoprim (15–20 mg/kg/day)/sulfamethoxazole (75–100 mg/kg/day), administered over 3–4 doses, infused over 1 h per day and duration of therapy is 21 days [42]. Oral TMP/SMX can be used for mild/moderate disease in the absence of gastrointestinal issues. The recommended second-line therapy in infants intolerant to TMP/SMX is IV pentamidine, if clinical improvement is evident after 7–10 days, a switch to oral pentamidine can be pursued for the remainder of the 3 weeks [42]. The other second line therapies have limited or no data for use in paediatrics. Short courses of corticosteroids are recommended for the management of PcP in HIV-positive children, but dose varies between studies [42].

Monitoring temperature, respiratory rate, arterial O_2_ saturation and chest radiology is recommended to determine response, which would be expected between 5–7 days, noting that deterioration can occur in the first 3-5 days. If there is no clinical improvement or worsening of symptoms after 4–8 days of therapy and other infections have been ruled-out, treatment failure should be considered. Side-effects to anti-PcP therapy have been noted, but are usually less frequent than in adults, usually reversible upon treatment cessation and rarely life threatening. [126] Re-challenging may be considered unless the preceding adverse event was severe. Secondary prophylaxis is required for all treated cases, following the guidance for primary prophylaxis. Intensive investigations are required should typical symptoms return post prophylaxis.

PcP in HIV-negative at-risk paediatric patients is less well studied. Connective tissue disease, haematological malignancy, kidney disease, and immunodeficiency increase the risk of PcP [127]. Mortality is high (>40%), and is, in the absence of PcP prophylaxis, significantly associated with the use of corticosteroids. Elevated LDH, mechanical ventilation and co-infection increase mortality. The CD4/CD8 ratio has been used a biomarker for predicting and diagnosing PcP in HIV-negative infants [127].

## 10. Concluding Remarks

The incidence of PcP is increasing in an ever-diverse at-risk population, which will continue to expand with advancements in clinical care. While guidelines are available for the management of well recognized at-risk cohorts, there is a growing need for guidance in other patient populations. In these populations, the absence of PcP prophylaxis, due to limited awareness of risk, may be detrimental to the individual and could result in outbreak scenarios. Infection control guidance for the management of outbreaks is also required. TMP/SMX is the drug of choice for both prophylaxis and therapy, across all at-risk groups, including paediatrics. The sequencing of the *Pneumocystis* genome should be used to identify novel targets for the treatment of PcP, which is particularly important with the emergence of resistant disease [128]. The availability of BDG and PCR tests will likely enhance diagnosis. Molecular diagnostics may also play a vital role in identifying resistant PcP cases, although the impact of mutations in the genes encoding proteins targeted by anti-PCP therapy is not yet clear, but is of concern. The scale of resistant disease is difficult to accurately determine, with most centres lacking the ability to identify resistance. Currently, intolerance to treatment is likely more problematic in the management of PcP, but this could change if the incidence of resistance is more precisely determined through the widespread introduction of novel molecular technologies, which also aid outbreak investigations.

## Figures and Tables

**Table 1:** A selection of *Pneumocystis* Pneumonia cases involving patients with less typical underlying conditions.

Underlying Disease	Number of Cases/ Medical History	Treatment	Outcome	Reference
Severe alcoholic hepatitis	2	Not available	Both died	[10]
Giant cell arteritis	7 F = 5 M = 2	All on prednisone at diagnosis of PcP No PcP prophylaxis All received trimethoprim/sulfamethoxazole for PcP	5 recovered 2 died	[8]
Crohn’s disease.	1 M, 19 y Post-marketing surveillance through June 2001, ntified 10 cases of PcP during infliximab treatment, 3 of which died	For Crohn’s disease— Azathioprine and infliximab For PcP— Steroids and trimethoprim-sulfamethoxazole	Follow up 2 weeks later confirmed clinical response to therapy.	[11]
Pustular psoriasis with an IL-36RN deficiency.	1 M, 54 y PCP developed after infliximab	For pustular psoriasis— Cyclosporin which was unresponsive then Infliximab For PcP IV trimethoprim/ sulfamethoxazole	Mutation found in the IL36RN gene compatible with IL-36RN deficiency anakinra started and psoriasis improved	[12]
Diabetes mellitus with pneumoconiosis and interstitial pneumonia	1 M, 75 y Carcinoma of the buccal mucosa	Corticosteroids for interstitial pneumonia Trimethoprim/sulfamethoxazole and voriconazole for PcP and *Aspergillus fumigatus*	Died	[13]
Systemic lupus erythematosus.	5 cases Study of 858 hospitalized lupus patients. ID from lung biopsy in 2 and BAL in 3	Prednisolone and concomitant biologics or immunosuppressants	3 died	[9]
Hyper-IgM syndrome	1 M, 4 months	IgM syndrome diagnosis made after PcP was detected. trimethoprim/sulfamethoxazole for PcP	Recovered	[14]
Membranoproliferative glomerulonephritis	1 M, 50 y	Corticosteroids for underlying condition For PcP Trimethoprim/sulfamethoxazole	Recovered	[15]
Dermatomyositis associated with anti-MDA-5 autoanti bodies	2 M, 56 y Initially had interstitial lung disease without infection M, 52 y	Both treated with corticosteroids for underlying condition. Both had specific treatment for PcP M, 52 y also received cyclophosphamide bolus	Both died	[16]
Cushing’s Syndrome	15 13 developed PcP after initiation of cortisol blocking therapy.	Cushing’s syndrome—Cortisol blocking therapy PcP therapy—Not stated	11 of the 15 patients died	[17]
*Pneumocystis jiroveci* pneumonia after infliximab therapy: a review of 84 cases.	84 Cases		23 of the 84 patients died	[18]

M: Male; F: Female; y: years; BAL: Bronchoalveolar lavage fluid; IV: Intravenous.

**Table 2:** Classification of *Pneumocystis* pneumonia [19].

Clinical factor	Disease Classification		
	Mild	Moderate	Severe
Dysnoea	On exertion	On minimal exertion/possibly at rest	At rest
Resting arterial tension	PaO_2_ of > 11.0 kPa	PaO_2_ 8.1–11.0 kPa	PaO_2_ < 8.0 kPa
Oxygen saturation	SaO_2_ > 96%	SaO_2_ of 91–96%	SaO_2_ < 91%
Radiology	Normal/Minimal changes on CXR	Diffuse interstitial changes on CXR	Extensive interstitial changes with potential diffuse alveolar shadowing on CXR
Other		Possibly Fever	Tachypnoea at rest, fever, cough

PaO_2_: Partial pressure of oxygen in blood; SaO_2_: Oxygen saturation; CXR: Chest radiograph.

**Table 3:** A selection of *Pneumocystis* Pneumonia cases involving co-infection with other pathogens.

Underlying Disease	Coinfection	Number of Cases/Medical History	Treatment	Outcome	Reference
COPD and chronic hepatitis C	PcP and Aspergillosis	1 M, 95 y, treated for lung injury caused by *Chlamydophila* pnemonia	Ceftriaxone and Methylprednisolone Then alternating prednisolone and methylprednisolone Then meropenem and azithromycin. Then sulfamethoxazole/ trimethoprim for PcP Then levofloxacin, minocycline, liposomal amphotericin B along with PcP treatment	Died of multiple organ failure	[24]
Crescentic IgA nephropathy and Non-Hodgkin lymphoma	PcP and Aspergillosis	2 M, 29 y with crescentic IgA nephropathy on immunosuppressive drugs F, 72 y with non-Hodgkin lymphoma on chemotherapy	Both— Intravenous trimethroprim/sulphamethoxazole combined with voriconazole Prophylaxis M—Moxifloxacin and Ganciclovir F—Moxifloxacin and Valaciclovir	M—Recovered F—Died	[25]
Alcoholic hepatitis and cirrhosis	PcP and cytomegalovirus	1 M, 40 y	Initially broad spectrum antibiotics then prednisolone. Amphotericin B syrup dissolved in water gargled for oral and esophageal candidiasis No treatment for PCP, found during autopsy	Died of circulatory insufficiency	[26]
Allopurinol-induced Stevens-Johnson syndrome and toxic epidermal necrolysis	PcP, parainfluenza virus type 3, CMV and *Aspergillus fumigatus*.	1 F, 63 y Presented with mucocutaneous macular skin rash with bulla. 3 months prior had an acute myocardial infarction. 1-month prior essential thrombocytosis 30 years previous had, surgery for intestinal TB and was a hepatitis C carrier.	High-dose systemic corticosteroids and intravenous immunoglobulin for Stevens-Johnson syndrome / toxic epidermal necrolysis 100 mg/day aspirin 1 mg/day anagrelide 200 mg/day allopurinol Anti-TB treatment 30 years ago. For co-infections Trimethroprim/sulphamethoxazole, voriconazole, ganciclovir and oral ribavirin.	Recovered	[27]
Probable plasma cell myeloma	Streptococcal meningitis and PcP	1 F, 64 y	Intravenous acyclovir, ceftriaxone and fluconazole for the meningitis Trimethroprim-sulfamethoxazole for PcP	Unclear	[28]
HTLV-1 Associated Adult T-cell leukemia/lymphoma	PcP and *Cryptococcus neoformans*	1	Trimethroprim-sulfamethoxazole, corticosteroids and fluconazole	Recovered	[29]
HIV	PcP and *Strongyloides stercoralis*	1 M, 43 y	Trimethroprim-sulfamethoxazole, ivermectin and amphotericin B	Died	[30]
Allogeneic hematopoietic stem cell transplantation recipient	PcP and Influenza A	1 M, 53 y Immunosuppresive therapy for treatment of GVHD	For underlying condition - daily mycophenolate mofetil, tacrolimus and prophylactic Trimethroprim-sulfamethoxazole. Treated with high-dose corticosteroids for GVHD PCP prophylaxis switched to inhaled pentamidine due to pancytopenia attributed to Trimethroprim-sulfamethoxazole. PcP treated with Trimethroprim-sulfamethoxazole.	Recovered	[31]
Infantile spasm	*Legionella pneumophila* and PcP	1 8 month old infant	Not Stated	Died	[32]
HIV	*Mycobacterium tuberculosis* and PcP	1	Trimethroprim-sulfamethoxazole prophylaxis for PcP Antituberculosis medication and Trimethroprim-sulfamethoxazole	Recovered	[33]

COPD: Chronic obstructive pulmonary disorder; GVHD: Graft versus host disease; TB: *Mycobacterium tuberculosis.*

**Table 4:** Prophylactic and therapeutic options for the management of *Pneumocystis* pneumonia in HIV, haematology, and solid organ transplant recipients.

Strategy	Population [Ref]		
	HIV-Positive [39]	Haematology [20,43]	Solid Organ Transplantation [44]
Prophylaxis	Front line: Trimethoprim/sulfamethoxazole one single-strength (80 mg TMP/400 mg SMX) daily or one double strength tablet (160 mg TMP/800 mg SMX)/daily. Second line: Trimethoprim/sulfamethoxazole one double strength tablet (160 mg TMP/800 mg SMX) three times per week Dapsone (50 mg twice daily) Dapsone (200 mg) + pyrimethamine (75 mg) + leucovorin (25 mg) weekly Dapsone (50 mg daily) + pyrimethamine (50 mg weekly) + leucovorin (25 mg weekly) Pentamidine aerosols (300 mg per month) Atovaquone 1500 mg daily	Front line: Trimethoprim/sulfamethoxazole one single-strength (80 mg TMP/400 mg SMX)/day or double strength tablet (160 mg TMP/800 mg SMX)/day or three per week. Second line: Dapsone (50 mg twice daily) Pentamidine aerosols (300 mg per month) Atovaquone (1500 mg daily)	Front line: Trimethoprim/sulfamethoxazole one single-strength (80 mg TMP/400 mg SMX)/day or double strength tablet (160 mg TMP/800 mg SMX)/day or three per week. Second line: Dapsone (50–100 mg once a day) Atovaquone (>1000 mg daily) Third Line: Pentamidine aerosols (300 mg every 3–4 weeks)
Treatment	Frontline: Trimethoprim/sulfamethoxazole (15–20 mg/kg TMP; 75–100 mg/kg SMX per day) For moderate to severe disease (i.e., hypoxemia) adjunctive corticosteroids should be used Second line for severe disease: Primaquine and clindamycin (30 mg/(600 mg × 3)) per day Pentamidine IV (4 mg/kg/day) Second line for mild/moderate disease: Dapsone (100 mg daily) + trimethoprim (15 mg daily) Atovaquone (750 mg BID)	Frontline: Trimethoprim/sulfamethoxazole (15–20 mg/kg TMP; 75–100 mg/kg SMX per day) Second line: Primaquine and clindamycin (30 mg/(600 mg × 3)) per day Pentamidine IV (4 mg/kg/day) Atovaquone (750 mg/ 2–3 per day)	Frontline: Trimethoprim/sulfamethoxazole (15–20 mg/kg TMP; 75–100 mg/kg SMX per day) with TMP administered by IV every 6–8 h. For hypoxemic patients potentially in combination with 40–60 mg of prednisolone (twice daily) Second line: IV Pentamidine (Initially 4 mg/kg/day over 1–2 h) Recipients of pancreas/islet transplants should receive an alternative second line therapy.

IV: Intravenous.

**Table 5:** A selection of clinical cases reporting resistant *Pneumocystis pneumonia.*

Underlying Disease	Number of Cases/ Medical History	Mechanism of Resistance	Alternative Treatment	Outcome	Reference
HIV	1 M, 50 y	Mutations at codons 55 and 57 of the, associated with resistance to Trimethoprim/sulfamethoxazole.	Clindamycin-primaquine	Recovered	[103]
HIV	13 M = 11 F = 2 Each patient had 2 episodes	3 patients had no PCP prophylaxis for both episodes only one had PcP mutations. 4 patients had no DHPS genotype mutations in their Positive PcP samples 9 patients had at least one of the mutations described below in their PcP samples M1, mutant 1 (Ala55 Pro57); M2, mutant 2 (Thr55 Ser57); M3, mutant 3 (Ala55 Ser57 double mutant).	Of the 11 patients who recovered, 9 received prophylaxis and all needed alternative therapy.	1 with no prophylaxis died on Trimethoprim/sulfamethoxazole treatment, PcP mutations present. 1 with prophylaxis on 2nd presentation died after alternative therapy of Trimethoprim/sulfamethoxazole then pentamidine only had wild type PcP. 11 recovered.	[104]
HIV	152	31 of the 152 had *Pnuemocystis carinii* DHPS mutations These mutations were more common in patients who had previous exposure to sulpha drugs	Not available	Survival of patients with mutations was significantly lowered	[105]
HIV and Non-HIV Immuno-suppressed	56 46 HIV 10 Non-HIV Immuno-suppressed	Mutations in the DHPS gene	Not available	All HIV patients recovered 5 of the 10 Non-HIV patients died	[106]
HIV	1 M, 50 y No previous exposure to Trimethoprim/sulfamethoxazole	DHPS gene mutations at codon 55 and 57 (Thr55Ala and Pro57Ser)	Clindamycin-primaquine	Recovered	[107]
Non-HIV Immunocompromised Patients	18	Substitution mutations: At DHPS codon 98—glutamate replaced by lysine. At DHPS codon 90—aspartate replaced by asparagine.	All patients received immunosuppressive agents but none of them received PcP prophylaxis For PcP— Trimethoprim/sulfamethoxazole Trimethoprim/sulfamethoxazole + Caspofungin Primaquine + clindamycin	Approximately 65% mortality	[108]
HIV	8	Cytochrome b substitutions in the Qo region: T121I, L123F, T100I, I120V, S125A, P239L and L248F	5 patients received Atovaquone prophylaxis, but 3 were Atovaquone naïve	84% overall survival: 87% of patients with mutations survived	[76]

DHPS: dihydropteroate synthase gene.

## References

[1] Maini R., Henderson K.L., Sheridan E.A., Lamagni T., Nichols G., Delpech V., Phin N. (2013). Increasing *Pneumocystis* pneumonia, England, UK, 2000–2010. Emerg. Infect. Dis..

[2] Patterson L., Coyle P., Curran T., Verlander N.Q., Johnston J. (2017). Changing epidemiology of *Pneumocystis* pneumonia, Northern Ireland, UK and implications for prevention, 1 July 2011–31 July 2012. J. Med. Microbiol.

[3] Pegorie M., Denning D.W., Welfare W. (2017). Estimating the burden of Invasive and Serious Fungal Disease in the United Kingdom. J. Infect..

[4] Buchacz K., Lau B., Jing Y., Bosch R., Abraham A.G., Gill M.J., Silverberg M.J., Goedert J.J., Sterling T.R., Althoff K.N. (2016). Incidence of AIDS-Defining Opportunistic Infections in a Multicohort Analysis of HIV-infected Persons in the United States and Canada, 2000–2010. J. Infect. Dis..

[5] Williams K.M., Ahn K.W., Chen M., Aljurf M.D., Agwu A.L., Chen A.R., Walsh T.J., Szabolcs P., Boeckh M.J., Auletta J.J. (2016). The incidence, mortality and timing of *Pneumocystis jiroveci* pneumonia after hematopoietic cell transplantation: A CIBMTR analysis. Bone Marrow Transplant..

[6] Avino L.J., Naylor S.M., Roecker A.M. (2016). Pneumocystis jirovecii pneumonia in the non-HIV infected population. Ann. Pharmacother..

[7] Iriat X., Challan Belval T., Fillaux J., Esposito L., Lavergne R.A., Cardeau-Desangles I., Roques O., Del Bello A., Cointault O., Lavayssière L. (2015). Risk factors of *Pneumocystis* Pneumonia in Solid Organ recipients in the era of the common use of post transplantation prophylaxis. Am. J. Transplant..

[8] Kermani T., Ytterberg S., Warrington K. (2011). *Pneumocystis jiroveci* pneumonia in giant cell arteritis: A case series. Arthritis Care Res..

[9] Weng C., Liu M., Weng M., Lee N., Wang M., Lin W., Ou C., Lai W., Hsu S., Chao S. (2013). *Pneumocystis jirovecii* Pneumonia in Systemic Lupus Erythematosus from Southern Taiwan. J. Clin. Rheumatol..

[10] Ichai P., Azoulay D., Feray C., Saliba F., Antoun F., Roche B., Bismuth H., Samuel D. (2002). *Pneumocystis carinii* and cytomegalovirus pneumonia after corticosteroid therapy in acute severe alcoholic hepatitis: 2 case reports. Gastroenterol. Clin. Biol..

[11] Velayos F., Sandborn W. (2004). *Pneumocystis carinii* Pneumonia During Maintenance Anti-Tumor Necrosis Factor-α Therapy with Infliximab for Crohn’s Disease. Inflamm. Bowel Dis..

[12] Podlipnik S., de la Mora L., Alsina M., Mascaró J. (2016). *Pneumocystis jirovecii* pneumonia in a patient with pustular psoriasis with an IL-36RN deficiency treated with infliximab: Case report and review of the literature. Australas. J. Dermatol..

[13] Mukasa Y., Ichiyasu H., Akaike K., Okamoto S., Komohara Y., Kohrogi H. (2010). Autopsy case of pulmonary zygomycosis and *Pneumocystis* pneumonia in a patient with interstitial pneumonia treated by corticosteroid therapy. Nihon Kokyuki Gakkai Zasshi.

[14] Heiming R., Beuschausen T., Liebner T., Seidenberg J., Roesler J. (1993). Life threatening *Pneumocystis carinii* pneumonia in a 4-month-old boy with hyper-IgM syndrome. Monatsschr Kinderheilkd.

[15] Plakke M., Jalota L., Lloyd B. (2013). *Pneumocystis* pneumonia in a non-HIV patient on chronic corticosteroid therapy: A question of prophylaxis. Case Rep..

[16] Aymonier M., Abed S., Boyé T., Barazzutti H., Fournier B., Morand J. (2017). Dermatomyosite associée aux anticorps anti-MDA5 et pneumocystose pulmonaire: Deux cas d’évolution fatale. Ann. Dermatol. Venereol..

[17] Van Halem K., Vrolijk L., Pereira A.M., de Boer M.G.J. (2017). Characteristics and Mortality of *Pneumocystis* Pneumonia in patients with Cushing’s syndrome: A Plea for timely initiation of chemoprophylaxis. Open Forum Infect. Dis..

[18] Kaur N., Mahl T. (2007). *Pneumocystis jiroveci* (*carinii*) Pneumonia After Infliximab Therapy: A Review of 84 Cases. Dig. Dis. Sci..

[19] Miller R.F., Le Noury J., Corbett E.L., Felton J.M., De Cock K.M. (1996). *Pneumocystis carinii* infection: Current treatment and prevention. J. Antimicrob. Chemother..

[20] Maschmeyer G., Helweg-Larsen J., Pagano L., Robin C., Cordonnier C., Schellongowski P. (2016). 6th European Conference on Infections in Leukemia (ECIL-6), a joint venture of The European Group for Blood and Marrow Transplantation (EBMT), The European Organization for Research and Treatment of Cancer (EORTC), the International Immunocompromised Host Society (ICHS) and The European LeukemiaNet (ELN). ECIL guidelines for treatment of *Pneumocystis jirovecii* pneumonia in non-HIV-infected haematology patients. J. Antimicrob. Chemother..

[21] White P.L., Backx M., Barnes R.A. (2017). Diagnosis and management of *Pneumocystis jirovecii* infection. Expert Rev. Anti. Infect. Ther.

[22] Iriart X., Bouar M.L., Kamar N., Berry A. (2015). *Pneumocystis* Pneumonia in Solid-Organ Transplant Recipients. J. Fungi.

[23] Liu Y., Su L., Jiang S.J., Qu H. (2017). Risk factors for mortality from *Pneumocystis carinii* pneumonia (PCP) in non-HIV patients: A meta-analysis. Oncotarget.

[24] Hagiya H., Kuroe Y., Nojima H., Sugiyama J., Naito H., Hagioka S., Morimoto N., Miyake T., Kokumai Y., Murase T. (2013). Co-infection with invasive pulmonary aspergillosis and *Pneumocystis jirovecii* pneumonia after corticosteroid therapy. J. Infect. Chemother..

[25] Markantonatou A., Ioakimidou A., Arvaniti K., Manou E., Papadopoulos V., Kiriklidou P., Samaras K., Kioumi A., Vyzantiadis T. (2017). Pulmonary co-infections by *Pneumocystis jirovecii* and Aspergillus fumigatus in non-HIV patients: A report of two cases and literature review. Mycoses.

[26] Ikawa H., Hayashi Y., Ohbayashi C., Tankawa H., Itoh H. (2001). Autopsy case of alcoholic hepatitis and cirrhosis treated with corticosteroids and affected by *Pneumocystis carinii* and cytomegalovirus pneumonia. Pathol. Int.

[27] Kim T., Lee T., Bae Y., Park S., Park H., Kim S., Cho Y., Moon H., Lee S. (2010). Severe Pneumonia Caused by Combined Infection with *Pneumocystis jiroveci*, Parainfluenza Virus Type 3, Cytomegalovirus, and Aspergillus fumigatus in a Patient with Stevens-Johnson Syndrome/toxic Epidermal Necrolysis. Acta Derm. Venereol..

[28] Dunphy L., Singh N., Keating E. (2017). Multiple myeloma presenting with bilateral ankle pain (microangiopathy) and complicated by streptococcal meningitis and *Pneumocystis carinii* pneumonia. BMJ Case Rep..

[29] Desai A., Fe A., Desai A., Ilowite J., Cunha B., Mathew J. (2016). A Case of Pneumonia Caused by *Pneumocystis jirovecii* and Cryptococcus Neoformans in a Patient with HTLV-1 Associated Adult T-Cell Leukemia/Lymphoma: Occam’s Razor Blunted. Conn. Med..

[30] Bava A., Romero M., Prieto R., Troncoso A. (2011). A case report of pulmonary coinfection of *Strongyloides stercoralis* and *Pneumocystis jiroveci*. Asian Pac. J. Trop. Biomed..

[31] Burke J., Soubani A. (2017). Influenza and *Pneumocystis jirovecii* pneumonia in an allogeneic hematopoietic stem cell transplantation recipient: Coinfection or superinfection?. Transpl. Infect. Dis..

[32] Musallam N., Bamberger E., Srugo I., Dabbah H., Glikman D., Zonis Z., Kessel A., Genizi J. (2013). Legionella pneumophila and *Pneumocystis jirovecii* Coinfection in an Infant Treated with Adrenocorticotropic Hormone for Infantile Spasm. J. Child Neurol.

[33] Khawcharoenporn T., Apisarnthanarak A., Sakonlaya D., Mundy L., Bailey T. (2006). Dual Infection with Mycobacterium tuberculosis and *Pneumocystis jiroveci* Lymphadenitis in a Patient with HIV Infection: Case Report and Review of the Literature. AIDS Patient Care STDS.

[34] Alvaro-Meca A., Palomares-Sancho I., Diaz A., Resino R., De Miguel G., Resino S. (2015). *Pneumocystis* pneumonia in HIV-positive patients in Spain: Epidemiology and environmental risk factors. J. Int. AIDS Soc..

[35] Sing A., Schmoldt S., Laubender R.P., Heesemann J., Sing D., Wildner M. (2009). Seasonal variation of *Pneumocystis jirovecii* infection: Analysis of underlying climatic factors. Clin. Microbiol. Infect..

[36] Miller R.F., Evans H.E., Copas A.J., Huggett J.F., Edwards S.G., Walzer P.D. (2010). Seasonal variation in mortality of *Pneumocystis jirovecii* pneumonia in HIV-infected patients. Int. J. STD AIDS.

[37] Fei M.W., Kim E.J., Sant C.A., Jarlsberg G.L., Davis J.L., Swartzman A., Huang L. (2009). Prediciting mortality from HIV-associated *Pneumocystis* pneumonia at illness presentation: An observational cohort study. Thorax.

[38] Weng L., Huang X., Chen L., Feng L.Q., Jiang W., Hu X.Y., Peng J.M., Wang C.Y., Zhan Q.Y., Du B. (2016). Prognostic factors for severe *Pneumocystis jiroveci* pneumonia of non-HIV patients in intensive care unit: A bicentric retrospective study. BMC Infect. Dis..

[39] Guidelines for the Prevention and Treatment of Opportunistic Infections in HIV-Infected Adults and Adolescents. http://aidsinfo.nih.gov/contentfiles/lvguidelines/adult_oi.pdf.

[40] Cordonnier C., Cesaro S., Maschmeyer G., Einsele H., Donnelly J.P., Alanio A., Hauser PM., Lagrou K., Melchers W.J., Helweg-Larsen J. (2016). Fifth European Conference on Infections in Leukemia (ECIL-5), a joint venture of The European Group for Blood and Marrow Transplantation (EBMT), The European Organization for Research and Treatment of Cancer (EORTC), the Immunocompromised Host Society (ICHS) and The European LeukemiaNet (ELN). *Pneumocystis jirovecii* pneumonia: Still a concern in patients with haematological malignancies and stem cell transplant recipients. J. Antimicrob. Chemother..

[41] Alanio A., Hauser P.M., Lagrou K., Melchers W.J., Helweg-Larsen J., Matos O., Cesaro S., Maschmeyer G., Einsele H., Donnelly J.P. (2016). 5th European Conference on Infections in Leukemia (ECIL-5), a joint venture of The European Group for Blood and Marrow Transplantation (EBMT), The European Organization for Research and Treatment of Cancer (EORTC), the Immunocompromised Host Society (ICHS) and The European LeukemiaNet (ELN). ECIL guidelines for the diagnosis of *Pneumocystis jirovecii* pneumonia in patients with haematological malignancies and stem cell transplant recipients. J. Antimicrob. Chemother..

[42] Guidelines for the Prevention and Treatment of Opportunistic Infections in HIV-Exposed and HIV-Infected Children. https://aidsetc.org/disclaimer.

[43] Maertens J., Cesaro S., Maschmeyer G., Einsele H., Donnelly J.P., Alanio A., Hauser P.M., Lagrou K., Melchers W.J., Helweg-Larsen J. (2016). 5th European Conference on Infections in Leukaemia (ECIL-5), a joint venture of the European Group for Blood and Marrow Transplantation (EBMT), the European Organisation for Research and Treatment of Cancer (EORTC), the Immunocompromised Host Society (ICHS) and the European LeukemiaNet (ELN). ECIL guidelines for preventing *Pneumocystis jirovecii* pneumonia in patients with haematological malignancies and stem cell transplant recipients. J. Antimicrob. Chemother..

[44] Martin S.I., Fishman J.A., The AST Infectious Diseases Community of Practice (2013). *Pneumocystis* pneumonia in Solid organ transplantation. Am. J. Translant..

[45] Mu X.-D., Jia P., Gao Li., Su L., Zhang C., Wang R.-G., Wang G.-F. (2016). Relationship between radiological stages and prognoses of *Pneumocystis* pneumonia in Non-AIDS immunocompromised patients. J. Chin. Med..

[46] Vogel M.N., Brodoefel H., Hierl T., Beck R., Bethge W.A., Claussen C.D., Horger M.S. (2007). Differences and similarities of cytomegalovirus and *Pneumocystis* pneumonia in HIV-negative immunocompromised patients thin section CT morphology in the early phase of the disease. Br. J. Radiol..

[47] Salzer H.J.F., Schäfer G., Hoenigl M., Günther G., Hoffmann C., Kalsdorf B., Alanio A., Lange C. (2018). Clinical, Diagnostic, and Treatment Disparities between HIV-Infected and Non-HIV-Infected Immunocompromised Patients with *Pneumocystis jirovecii* Pneumonia. Respiration.

[48] Vogel M.N., Weissgerber P., Goeppert B., Hetzel J., Vatlach M., Claussen C., Horger M. (2011). Accuracy of serum LDH elevation for the diagnosis of *Pneumocystis jiroveci* pneumonia. Swiss Med Wkly.

[49] Nyamande K., Lalloo U.G. (2006). Serum procalcitonin distinguishes CAP due to bacteria, Mycobacterium tuberculosis and PJP. Int. J. Tuberc. Lund Dis..

[50] Schildgen V., Mai S., Khalfaoui S., Lüsebrink J., Pieper M., Tillmann R.L., Brockmann M., Schildgen O. (2014). *Pneumocystis jiroveci* can be productively cultured in differentiated CuFi-8 airway cells. Mbio.

[51] Cruciani M., Marcati P., Malena M., Bosco O., Serpelloni G., Mengoli C. (2002). Meta-analysis of diagnostic procedures for *Pneumocystis* carnii pneumonia in HIV-1-infected patients. Eur. Respir. J..

[52] Summah H., Zhu Y.-G., Falagas M.E., Vouloumanou E.K., Qu J.-M. (2013). Use of real-time polymerase chain reaction for the diagnosis of *Pneumocystis* pneumonia in immuncompromised patients: A meta-analysis. J. Chin. Med..

[53] Fan L.-C., Lu H.-W., Cheng K.-B., Li H.-P., Xu J.-F. (2013). Evaluation of PCR in bronchoalveolar lavage fluid for diagnosis of *Pneumocystis jirovecii* pneumonia: A bivariate meta-analysis and systematic review. PLoS ONE.

[54] Lu Y., Ling G., Qiang C., Ming Q., Wu C., Wang K., Ying Z. (2011). PCR Diagnosis of *Pneumocystis* pneumonia: A bivariate meta-analysis. J. Clin. Microbiol..

[55] Sasso M., Chastang-Dumas E., Bastide S., Alonso S., Lechiche C., Bourgeois N., Lachaud L. (2016). Performances of four real-time PCR assays for the diagnosis of *Pneumocystis jirovecii* Pneumonia. J. Clin. Microbiol..

[56] Karageorgopoulos D.E., Qu J.M., Korbila I.P., Zhu Y.G., Vasileiou V.A., Falagas M.E. (2013). Accuracy of β-d-glucan for the diagnosis of *Pneumocystis jirovecii* pneumonia: A meta-analysis. Clin. Microbiol. Infect..

[57] Onishi A., Sugiyama D., Kogata Y., Saegusa J., Sugimoto T., Kawano S., Morinobu A., Nishimura K., Kumagai S. (2012). Diagnostic Accuracy of Serum 1,3-β-d-Glucan for *Pneumocystis jiroveci* Pneumonia, Invasive Candidiasis, and Invasive Aspergillosis: Systematic Review and Meta-Analysis. J. Clin. Microbiol..

[58] Li W.J., Guo Y.L., Liu T.J., Wang K., Kong J.L. (2015). Diagnosis of *Pneumocystis* pneumonia using serum (1-3)-β-d-Glucan:a bivariate meta-analysis and systematic review. Thorac. Dis..

[59] Damiani C., Le Gal S., Da Costa C., Virmaux M., Nevez G., Totet A. (2013). Combined quantification of pulmonary *Pneumocystis jirovecii* DNA and serum (1-3)-β-d-glucan for differential diagnosis of *Pneumocystis* pneumonia and *Pneumocystis* colonisation. J. Clin. Microbiol..

[60] Rose S.R., Vallabhajosyula S., Velez M.G., Fedorko D.P., VanRaden M.J., Gea-Banacloche J.C., Lionakis M.S. (2014). The utility of bronchoalveolar lavage β-d-glucan testing for the diagnosis of invasive fungal infections. J. Infect..

[61] Salerno D., Mushatt D., Myers L., Zhuang Y., de la Rua N., Calderon E.J., Welsh D.A. (2014). Serum and BAL β-d-glucan for the diagnosis of *Pneumocystis* pneumonia in HIV positive patients. Respir. Med..

[62] White P.L., Wingard J.R., Bretagne S., Löffler J., Patterson T.F., Slavin M.A., Barnes R.A., Pappas P.G., Donnelly J. (2015). Aspergillus Polymerase Chain Reaction: Systematic Review of Evidence for Clinical Use in Comparison with Antigen Testing. Clin. Infect. Dis..

[63] Alvarez B., Arcos J., Fernandez-Guerrero M.L. (2011). Pulmonary infectious diseases in patients with primary immunodeficiency and those treated with biologic immunomodulating agents. Curr. Opin. Pulm. Med..

[64] Baddley J.W., Winthrop K.L., Chen L., Liu L., Grijalva C.G., Delzell E., Beukelman T., Patkar N.M., Xie F., Saag K.G. (2014). Non-viral opportunistic infections in new users of tumour necrosis factor inhibitor therapy: Results of the SAfety Assessment of Biologic ThERapy (SABER) Study. Ann Rheum. Dis..

[65] Park J.W., Curtis J.R., Moon J., Song Y.W., Kim S., Lee E.B. (2018). Prophylactic effect of trimethoprim-sulfamethoxazole for *Pneumocystis* pneumonia in patients with rheumatic diseases exposed to prolonged high-dose glucocorticoids. Ann. Rheum. Dis.

[66] Green H., Paul M., Vidal L., Leibovici L. (2007). Prophylaxis of *Pneumocystis* pneumonia in immunocompromised non-HIV-infected patients: Systematic review and meta-analysis of randomized controlled trials. Mayo Clin. Proc..

[67] The Opportunistic Infections Project Team of the Collaboration of Observational HIV Epidemiological Research in Europe (COHERE) (2010). Is It Safe to Discontinue Primary *Pneumocystis jiroveci* Pneumonia Prophylaxis in Patients with Virologically Suppressed HIV Infection and a CD4 Cell Count <200 Cells/mL?. Clin. Infect. Dis..

[68] Fishman J.A. (2001). Prevention of infection caused by *Pneumocystis carinii* in transplant recipients. Clin. Infect. Dis..

[69] Kasiske B.L., Zeier M.G., Chapman J.R., Craig J.C., Ekberg H., Garvey C.A., Green M.D., Jha V., Josephson M.A., Kiberd B.A. (2010). KDIGO clinical practice guideline for the care kidney transplant recipients: A summary. Kidney Int..

[70] Goto N., Takahashi-Nakazato A., Futamura K., Okada M., Yamamoto T., Tsujita M., Hiramitsu T., Narumi S., Tsuchiya K., Gatanaga H. (2017). Lifelong Prophylaxis with Trimethoprim-Sulfamethoxazole for Prevention of Outbreak of *Pneumocystis jirovecii* Pneumonia in Kidney Transplant Recipients. Transplant. Direct..

[71] Faure E., Lionet A., Kipnis E., Noel C., Hazzan M. (2017). Risk factors for *Pneumocystis* pneumonia after the first 6 months following renal transplantation. Transplant. Infect. Dis..

[72] Stern A., Green H., Paul M., Vidal L., Leibovici L. (2014). Prophylaxis for *Pneumocystis* pneumonia (PCP) in non-HIV immunocompromised patients. Cochrane Database Syst. Rev..

[73] Evans R.A., Clifford T.M., Tang S., Au T., Fugit A.M. (2015). Efficacy of once-weekly dapsone dosing for *Pneumocystis jirovecii* pneumonia prophylaxis post transplantation. Transpl. Infect. Dis..

[74] Lee I., Barton T.D., Goral S., Doyle A.M., Bloom R.D., Chojnowski D., Korenda K., Blumberg E.A. (2005). Complications related to dapsone use for *Pneumocystis jirovecii* pneumonia prophylaxis in solid organ transplant recipients. Am. J. Transplant..

[75] Walker D.J., Wakefield A.E., Dohn M.N., Miller R.F., Baughman R.P., Hossler P.A., Bartlett M.S., Smith J.W., Kazanjian P., Meshnick S.R. (1998). Sequence polymorphisms in the *Pneumocystis carinii* cytochrome b gene and their association with atovaquone prophylaxis failure. J. Infect. Dis..

[76] Kazanjian P., Armstrong W., Hossler P.A., Lee C.H., Huang L., Beard C.B., Carter J., Crane L., Duchin J., Burman W. (2001). *Pneumocystis carinii* cytochrome b mutations are associated with atovaquone exposure in patients with AIDS. J. Infect. Dis..

[77] Argy N., Le Gal S., Coppée R., Song Z., Vindrios W., Massias L., Kao W.C., Hunte C., Yazdanpanah Y., Lucet J.C. (2018). *Pneumocystis* Cytochrome b Mutants Associated With Atovaquone Prophylaxis Failure as the Cause of *Pneumocystis* Infection Outbreak Among Heart Transplant Recipients. Clin. Infect. Dis..

[78] Vasconcelles M.J., Bernardo M.V., King C., Weller E.A., Antin J.H. (2000). Aerosolized pentamidine as *Pneumocystis* prophylaxis after bone marrow transplantation is inferior to other regimens and is associated with decreased survival and an increased risk of other infections. Biol. Blood Marrow Transplant..

[79] Souza J.P., Boeckh M., Gooley T.A., Flowers M.E.D., Crawford S.W. (1999). High rates of *Pneumocystis carinii* pneumonia in allogeneic blood and marrow transplant recipients receivingdapsone prophylaxis. Clin. Infect. Dis..

[80] Colby C., McAfee S., Sackstein R., Finkelstein D., Fishman J., Spitzer T. (1999). A prospective randomized trial comparing the toxicity and safety of atovaquone with SXTas *Pneumocystis carinii* pneumonia prophylaxis following autologous peripheral blood stem cell transplantation. Bone Marrow Transplant..

[81] Wolfe R.M., Peacock J.E. (2017). *Pneumocystis* Pneumonia and the Rheumatologist: Which Patients are at Risk and How Can PCP Be Prevented?. Curr. Rheumatol. Rep..

[82] Katsuyama T., Saito K., Kubo S., Nawata M., Tanaka Y. (2014). Prophylaxis for *Pneumocystis* pneumonia in patients with rheumatoid arthritis treated with biologics, based on risk factors found in a retrospective study. Arthritis Res. Ther..

[83] Baulier G., Issa N., Gabriel F., Accoceberry I., Camou F., Duffau P. (2018). Guidelines for prophylaxis of *Pneumocystis* pneumonia cannot rely solely on CD4-cell count in autoimmune and inflammatory diseases. Clin. Exp. Rheumatol..

[84] Lehman J.S., Gonzalez Santiago T.M., Wetter D.A., Kalaaji A.N., Limper A.H. (2017). Weighing the risks and benefits of *Pneumocystis* pneumonia prophylaxis in iatrogenically immunosuppressed dermatology patients. Int. J. Dermatol..

[85] Lawrence S.J., Sadarangani M., Jacobson K. (2017). Pneumocystis jirovecii Pneumonia in pediatric inflammatory bowel disease: A Case report and literature review. Front Pediatr..

[86] Cushion M.T., Collins M.S. (2011). Susceptibility of *Pneumocystis* to echinocandins in suspension and biofilm cultures. Antimicrob. Agents Chemother..

[87] Wang L.I., Liang H., Ye L.I., Jiang J., Liang B., Huang J. (2016). Adjunctive corticosteroids for the treatment of *Pneumocystis jiroveci* pneumonia in patients with HIV: A meta-analysis. Exp. Ther. Med..

[88] Zolopa A., Andersen J., Powderly W., Sanchez A., Sanne I., Suckow C., Hogg E., Komarow L. (2009). Early antiretroviral therapy reduces AIDS progression/death in individuals with acute opportunistic infections: A multicenter randomized strategy trial. PLoS ONE.

[89] Kosaka M., Ushiki A., Ikuyama Y., Hirai K., Matsuo A., Hachiya T., Hanaoka M. (2017). A Four-Center Retrospective Study of the Efficacy and Toxicity of Low-Dose Trimethoprim-Sulfamethoxazole for the Treatment of *Pneumocystis* Pneumonia in Patients without HIV Infection. Antimicrob. Agents Chemother..

[90] Bollee G., Sarfati C., Thiery G., Bergeron A., de Miranda S., Menotti J., de Castro N., Tazi A., Schlemmer B., Azoulay E. (2007). Clinical picture of *Pneumocystis jiroveci* pneumonia in cancer patients. Chest.

[91] Lemiale V., Debrumetz A., Delannoy A., Alberti C., Azoulay E. (2013). Adjunctive steroid in HIV-negative patients with severe *Pneumocystis* pneumonia. Respir. Res..

[92] Injean P., Eells S.J., Wu H., McElroy I., Gregson A.L., McKinnell J.A. (2017). A Systematic Review and Meta-Analysis of the Data Behind Current Recommendations for Corticosteroids in Non-HIV-Related PCP: Knowing When You Are on Shaky Foundations. Transplant. Direct..

[93] Wieruszewski P.M., Barreto J.N., Frazee E., Daniels C.E., Tosh P.K., Dierkhising R.A., Mara K.C., Limper AH. (2018). Early Corticosteroids for *Pneumocystis* Pneumonia in Adults Without HIV Are Not Associated with Better Outcome. Chest.

[94] Torres H.A., Chemaly R.F., Storey R., Aguilera E.A., Nogueras G.M., Safdar A., Rolston K.V., Raad I.I., Kontoyiannis D.P. (2006). Influence of type of cancer and hematopoietic stem cell transplantation on clinical presentation of *Pneumocystis jiroveci* pneumonia in cancer patients. Eur. J. Clin. Microbiol. Infect. Dis..

[95] Roger P.M., Vandenbos F., Pugliese P., De Salvador F., Durant J., Le Fichoux Y., Dellamonica P. (1998). Persistence of *Pneumocystis carinii* after effective treatment of *P. carinii* pneumonia is not related to relapse or survival among patients infected with human immunodeficiency virus infection. Clin. Infect. Dis..

[96] Monnet X., Vidal-Petiot E., Osman D., Hamzaoui O., Durrbach A., Goujard C., Miceli C., Bourée P., Richard C. (2008). Critical care management and outcome of severe *Pneumocystis* pneumonia in patients with and without HIV infection. Crit. Care.

[97] Soares M., Salluh J.I., Azoulay E. (2010). Noninvasive ventilation in patients with malignancies and hypoxemic acute respiratory failure: A still pending question. J. Crit. Care.

[98] Hardy W.D., Feinberg J., Finkelstein D.M., Power M.E., He W., Kaczka C., Frame P.T., Holmes M., Waskin H., Fass R.J. (1992). A controlled trial of trimethoprim-sulfamethoxazole or aerosolized pentamidine for secondary prophylaxis of *Pneumocystis carinii* pneumonia in patients with the acquired immunodeficiency syndrome. AIDS Clinical Trials Group Protocol 021. N. Engl. J. Med..

[99] Schneider M.M., Hoepelman A.I., Eeftinck Schattenkerk J.K., Nielen T.L., van der Graaf Y., Frissen J.P., van der Ende I.M., Kolsters A.F., Borleffs J.C. (1992). A controlled trial of aerosolized pentamidine or trimethoprim-sulfamethoxazole as primary prophylaxis against *Pneumocystis carinii* pneumonia in patients with human immunodeficiency virus infection. The Dutch AIDS Treatment Group. N. Engl. J. Med.

[100] Robin C., Lê M.P., Melica G., Massias L., Redjoul R., Khoudour N., Leclerc M., Beckerich F., Maury S., Hulin A. (2017). Plasma concentrations of atovaquone given to immunocompromised patients to prevent *Pneumocystis jirovecii*. J. Antimicrob. Chemother..

[101] Calderón M.M., Penzak S.R., Pau A.K., Kumar P., McManus M., Alfaro R.M., Kovacs J.A. (2016). Efavirenz but Not Atazanavir/Ritonavir Significantly Reduces Atovaquone Concentrations in HIV-Infected Subjects. Clin. Infect. Dis..

[102] Suárez I., Roderus L., van Gumpel E., Jung N., Lehmann C., Fätkenheuer G., Hartmann P., Plum G., Rybniker J. (2017). Low prevalence of DHFR and DHPS mutations in *Pneumocystis jirovecii* strains obtained from a German cohort. Infection.

[103] Lee S., Cho Y., Sung Y., Chung D., Jeong S., Park J., Lee S. (2015). A Case of Pneumonia Caused by *Pneumocystis jirovecii* Resistant to Trimethoprim-Sulfamethoxazole. Korean J. Parasitol..

[104] Nahimana A., Rabodonirina M., Helweg-Larsen J., Meneau I., Francioli P., Bille J., Hauser P. (2003). Sulfa Resistance and Dihydropteroate Synthase Mutants in Recurrent *Pneumocystis carinii* Pneumonia. Emerg. Infect. Dis..

[105] Helweg-Larsen J., Benfield T., Eugen-Olsen J., Lundgren J., Lundgren B. (1999). Effects of mutations in *Pneumocystis carinii* dihydropteroate synthase gene on outcome of AIDS-associated *P. carinii* pneumonia. Lancet.

[106] Ponce C.A., Chabé M., George C., Cárdenas A., Durán L., Guerrero J., Bustamante R., Matos O., Huang L., Miller R.F. (2017). High Prevalence of *Pneumocystis jirovecii* Dihydropteroate Synthase Gene Mutations in Patients with a First Episode of *Pneumocystis* Pneumonia in Santiago, Chile, and Clinical Response to Trimethoprim-Sulfamethoxazole Therapy. Antimicrob. Agents Chemother..

[107] Ahn A., Chang J., Sung H., Kim M. (2016). A Case of Pneumonia Caused by *Pneumocystis jirovecii* Resistant to SXT in the Absence of Previous Drug Exposure. Lab. Med. Online.

[108] Long Y., Zhang C., Su L., Que C. (2014). *Pneumocystis jirovecii* dihydropteroate synthase gene mutations in a group of HIV-negative immunocompromised patients with *Pneumocystis* pneumonia. Exp.Ther. Med..

[109] Hauser P.M., Nahimana A., Taffe P., Weber R., Francioli P., Bille J., Rabodonirina M. (2010). Interhuman transmission as a potential key parameter for geographical variation in the prevalence of *Pneumocystis jirovecii* dihydropteroate synthase mutations. Clin. Infect. Dis..

[110] Huang L., Beard C.B., Creasman J., Levy D., Duchin J.S., Lee S., Pieniazek N., Carter J.L., del Rio C., Rimland D., Navin T.R. (2000). Sulfa or sulfone prophylaxis and geographic region predict mutations in the *Pneumocystis carinii* dihydropteroate synthase gene. J. Infect. Dis..

[111] Montesinos I., Delforge M.L., Ajjaham F., Brancart F., Hites M., Jacobs F., Denis O. (2017). Evaluation of a new commercial real-time PCR assay for diagnosis of *Pneumocystis jirovecii* pneumonia and identification of dihydropteroate synthase (DHPS) mutations. Diagn. Microbiol. Infect. Dis..

[112] Yiannakis E.P., Boswell T.C. (2016). Systematic review of outbreaks of *Pneumocystis jirovecii* pneumonia: Evidence that *P. jirovecii* is a transmissible organism and the implications for healthcare infection control. J. Hosp. Infect..

[113] Miller R.F., Ambrose H.E., Wakefield A.E. (2001). *Pneumocystis carinii* f. sp. hominis DNA in immunocompetent health care workers in contact with patients with *P. carinii* pneumonia. J. Clin. Microbiol..

[114] Choukri F., Menotti J., Sarfati C., Lucet J.C., Nevez G., Garin Y.J., Derouin F., Totet A. (2010). Quantification and spread of *Pneumocystis jirovecii* in the surrounding air of patients with *Pneumocystis* pneumonia. Clin. Infect. Dis..

[115] De Boer M.G., Bruijnesteijn van Coppenraet L.E., Gaasbeek A., Berger S.P., Gelinck L.B., van Houwelingen H.C., van den Broek P., Kuijper E.J., Kroon F.P., Vandenbroucke J.P. (2007). An outbreak of *Pneumocystis jiroveci* pneumonia with 1 predominant genotype among renal transplant recipients: Interhuman transmission or a common environmental source?. Clin. Infect. Dis..

[116] Chapman J.R., Marriott D.J., Chen S.C., MacDonald P.S. (2013). Post-transplant *Pneumocystis jirovecii* pneumonia—A re-emerged public health problem?. Kidney Int..

[117] Bartlett M.S., Vermund S.H., Jacobs R., Durant P.J., Shaw M.M., Smith J.W., Tang X., Lu J.J., Li B., Jin S. (1997). Detection of *Pneumocystis carinii* DNA in air samples: Likely environmental risk to susceptible persons. J. Clin. Microbiol..

[118] Olsson M., Lidman C., Latouche S., Björkman A., Roux P., Linder E., Wahlgren M. (1998). Identification of *Pneumocystis carinii* f. sp. hominis gene sequences in filtered air in hospital environments. J. Clin. Microbiol..

[119] Mori S., Sugimoto M. (2012). *Pneumocystis jirovecii* infection: An emerging threat to patients with rheumatoid arthritis. Rheumatology.

[120] Yazaki H., Goto N., Uchida K., Kobayashi T., Gatanaga H., Oka S. (2009). Outbreak of *Pneumocystis jiroveci* pneumonia in renal transplant recipients: *P. jiroveci* is contagious to the susceptible host. Transplantation.

[121] Lee C.H., Helweg-Larsen J., Tang X., Jin S., Li B., Bartlett M.S., Lu J.J., Lundgren B., Lundgren J.D., Olsson M. (1998). Update on *Pneumocystis carinii* f. sp. hominis typing based on nucleotide sequence variations in internal transcribed spacer regions of rRNA genes. J. Clin. Microbiol..

[122] Lu J.J., Lee C.H. (2008). *Pneumocystis* pneumonia. J. Formos. Med. Assoc..

[123] Alanio A., Gits-Muselli M., Mercier-Delarue S., Dromer F., Bretagne S. (2016). Diversity of *Pneumocystis jirovecii* during Infection Revealed by Ultra-Deep Pyrosequencing. Front. Microbiol..

[124] Gits-Muselli M., Peraldi M.N., de Castro N., Delcey V., Menotti J., Guigue N., Hamane S., Raffoux E., Bergeron A., Valade S. (2015). New Short Tandem Repeat-Based Molecular Typing Method for *Pneumocystis jirovecii* Reveals Intrahospital Transmission between Patients from Different Wards. PLoS ONE.

[125] Mortier E., Pouchot J., Bossi P., Molinié V. (1995). Maternal-fetal transmission of *Pneumocystis carinii* in human immunodeficiency virus infection. N. Engl. J. Med..

[126] Schneider M.M., Borleffs J.C., Stolk R.P., Jaspers C.A., Hoepelman A.I. (1999). Discontinuation of prophylaxis for *Pneumocystis carinii* pneumonia in HIV-1-infected patients treated with highly active antiretroviral therapy. Lancet.

[127] Ling C., Qian S., Wang Q., Zeng J., Jia X., Liu J., Li Z. (2018). *Pneumocystis* pneumonia in non-HIV children: A 10-year retrospective study. J. Clin. Respir..

[128] Ma L., Chen Z., Huang da W., Kutty G., Ishihara M., Wang H., Abouelleil A., Bishop L., Davey E., Deng R. (2016). Genome analysis of three *Pneumocystis* species reveals adaptation mechanisms to life exclusively in mammalian hosts. Nat. Commun..

